# Structural and functional neural correlates of spatial navigation: a combined voxel‐based morphometry and functional connectivity study

**DOI:** 10.1002/brb3.572

**Published:** 2016-10-03

**Authors:** Xin Hao, Yi Huang, Xueting Li, Yiying Song, Xiangzhen Kong, Xu Wang, Zetian Yang, Zonglei Zhen, Jia Liu

**Affiliations:** ^1^ State Key Laboratory of Cognitive Neuroscience and Learning & IDG/McGovern Institute for Brain ResearchBeijing Normal UniversityBeijingChina; ^2^ Department of PsychologyTsinghua UniversityBeijingChina; ^3^ Department of PsychologyRenmin University of ChinaBeijingChina; ^4^ Beijing Key Laboratory of Applied Experimental PsychologySchool of PsychologyBeijing Normal UniversityBeijingChina

**Keywords:** cognitive map, heading direction, hierarchical brain network, scene perception, spatial navigation, voxel‐based morphometry

## Abstract

**Introduction:**

Navigation is a fundamental and multidimensional cognitive function that individuals rely on to move around the environment. In this study, we investigated the neural basis of human spatial navigation ability.

**Methods:**

A large cohort of participants (*N *> 200) was examined on their navigation ability behaviorally and structural and functional magnetic resonance imaging (MRI) were then used to explore the corresponding neural basis of spatial navigation.

**Results:**

The gray matter volume (GMV) of the bilateral parahippocampus (PHG), retrosplenial complex (RSC), entorhinal cortex (EC), hippocampus (HPC), and thalamus (THAL) was correlated with the participants’ self‐reported navigational ability in general, and their sense of direction in particular. Further fMRI studies showed that the PHG, RSC, and EC selectively responded to visually presented scenes, whereas the HPC and THAL showed no selectivity, suggesting a functional division of labor among these regions in spatial navigation. The resting‐state functional connectivity analysis further revealed a hierarchical neural network for navigation constituted by these regions, which can be further categorized into three relatively independent components (i.e., scene recognition component, cognitive map component, and the component of heading direction for locomotion, respectively).

**Conclusions:**

Our study combined multi‐modality imaging data to illustrate that multiple brain regions may work collaboratively to extract, integrate, store, and orientate spatial information to guide navigation behaviors.

## 1 Introduction

Being lost in a new city is troublesome, yet being lost in a hostile jungle was deadly for our ancestors. Successful spatial navigation, or environmental spatial cognition, is of enormous importance in our daily activities, such as finding the way back to a hotel or choosing alternative routes during rush hour. Though navigation is a fundamental cognitive ability, it is not a solitary function but instead consists of multiple cognitive components, including perceiving and integrating spatial information from multiple sensory modalities (e.g., visual and vestibular), forming and storing spatial representations (i.e., cognitive map), and retrieving and manipulating the spatial representations to guide navigation behaviors (Epstein & Vass, 2014; Montello, 2005; Wolbers & Hegarty, 2010).

Previous neuroimaging studies tend to design a variety of experimental paradigms to identify the neural basis for each specific cognitive component in navigation. The most classical design was to use house or scene stimuli to identify cortical regions involved in scene perception (see Epstein, 2008; for review; Troiani, Stigliani, Smith, & Epstein, 2012), such as parahippocampal place area (PPA, Epstein & Kanwisher, 1998), the retrosplenial complex (RSC, Bar & Aminoff, 2003; Maguire, 2001), and the transverse occipital sulcus (TOS, Hasson, Levy, Behrmann, Hendler, & Malach, 2002; Grill‐Spector, 2003; also known as the occipital place area, OPA, Dilks, Julian, Paunov, & Kanwisher, 2013). In addition, navigation tasks based on virtual environment were frequently adopted by fMRI studies, providing piles of evidence for a number of regions involved in specific component functions of navigation. For instance, the hippocampal system and striatal system are proposed to support boundary and landmark processing, respectively, in spatial learning (e.g., Doeller, King, & Burgess, 2008); the hippocampus (HPC) and RSC complementarily contribute to the formation and utilization of cognitive maps (Iaria, Chen, Guariglia, Ptito, & Petrides, 2007; Maguire et al., 1998; Wolbers & Buchel, 2005); the HPC and caudate nucleus subserve use of different navigation strategies (Iaria, Petrides, Dagher, Pike, & Bohbot, 2003); and the RSC anchors internal spatial representation to local environment (Marchette, Vass, Ryan, & Epstein, 2014).

However, these studies have two limitations. First, unlike small‐scale spatial cognition (e.g., mental rotation), navigation requires an individual's locomotion in a large‐scale environment. Navigation tasks based on virtual environments in the scanner simulate navigation behaviors in the real world (e.g., way‐finding in a large‐scale virtual space), and thus overcome this limitation to some extent. However, it has been proposed to be cautious on differences between authentic and simulated navigation behaviors (Montello, 2005; Montello & Sas, 2006; Taube, Valerio, & Yoder, 2013). Second, navigation ability is multifaceted (Weisberg, Schinazi, Newcombe, Shipley, & Epstein, 2013), while most previous fMRI studies focused on a specific component of spatial navigation and did not directly examine it as a whole.

To overcome the limitations, another line of study used the approach of individual differences to correlate behavioral performance in spatial navigation in a real environment with neural substrates of the brain. For example, individuals with extensive experiences in navigation (e.g., London taxi driver) have larger posterior hippocampal volume than normal adults (Maguire et al., 2000) or bus drivers (Maguire, Woollett, & Spiers, 2006). Importantly, the gray matter volume (GMV) of the posterior hippocampus is increased after training in spatial navigation (Woollett & Maguire, 2011). In addition, individuals with larger right posterior hippocampus and smaller GMV of the caudate are better at offsite pointing judgments (Schinazi, Nardi, Newcombe, Shipley, & Epstein, 2013; see also Bohbot, Lerch, Thorndycraft, Iaria, & Zijdenbos, 2007). A recent study correlated self‐reported measures on spatial navigation with both gray and white matter, and found that good navigators have higher GMV in right anterior parahippocampal and rhinal cortex, whereas bad navigators have higher GMV and fractional anisotropy (FA) values in or close to the caudate nucleus (Wegman et al., 2014).

In this study, we extended previous studies by asking a large cohort of participants (*N *= 298) to evaluate their navigation ability in general, in a real environment and then using VBM to identify as many cortical regions as possible that are related to spatial navigation across the whole brain. To further verify the functionality of the navigation‐related regions, we examined: (1) whether the GMV of these regions can predict participants’ sense of direction (Hegarty, Richardson, Montello, Lovelace, & Subbiah, 2002) and (2) how these regions responded to natural scenes when participants viewed movie clips in functional MRI scans. Finally, we explored the hierarchical structure of the network characterized by the resting functional connectivity (FC) among these regions. We predict that (1) neuroanatomical differences in the medial temporal lobe across the participants may underlie individual differences in self‐reported spatial navigation ability, and (2) the navigation‐related regions sharing similar cognitive processes are more likely to be clustered together than are regions involving in distinct functions (e.g., the parahippocampus [PHG] and RSC are clustered because of their role in coding scenes).

## 2 Methods

### 2.1 General procedure

Three imaging modalities (i.e., structural MRI, task‐state fMRI, and resting‐state FC) were combined to explore the neural basis of spatial navigation consecutively. First, the correlational analysis between GMV and navigation ability was performed in the VBM analysis, which identified regions that were associated with navigation ability. Second, the task‐state fMRI on scene perception was designated to examine whether the regions identified in the VBM analysis overlapped with the regions reported in previous fMRI studies, and to preliminarily differentiate the functionality of these regions by examining their activations in scene perception. Finally, if we observed functional division of labor of these regions in navigation, we performed a resting‐state FC analysis to examine the hypothesis that regions with a similar function shall be clustered in one subnetwork, which, along other subnetworks form a hierarchical structure of the navigation network. In short, the VBM analysis was used to identify navigation‐related regions, and the task‐state fMRI and resting‐state FC analyses were used to characterize the anatomy‐function correspondence and the hierarchical structure of these regions.

### 2.2 Participants

Two hundred and ninety‐eight college students (age: 20–25; mean = 23.32, *SD* = 0.83, 122 males) from Beijing Normal University, Beijing, China, participated in the study as a part of an ongoing project named GEB^2 (Gene Environment Brain & Behavior) (Huang et al., 2014; Kong, Hu, Xue, Song, & Liu, 2015; Kong et al., 2014; Song et al., 2014; Wang et al., 2014). Data that are irrelevant to the scope of this study were not reported here. Participants had no history of neurological disorders (e.g., mental retardation, traumatic brain injury) or psychiatric illnesses or current psychotropic medication use. In addition to structural MRI and resting fMRI scans, 30 participants (age: 22–25; 10 males) also participated in a task‐state fMRI scan. The experimental protocol was approved by the Institutional Review Board of Beijing Normal University. Written informed consent was obtained from all participants before the study.

### 2.3 Behavioral assessment of spatial cognition

#### 2.3.1 General navigation ability

Participants were asked to rate their own general navigational ability in a self‐report question, “Compared with your peers, how good is your spatial navigation ability?” The statement was ranked in a 5‐point Likert‐type scale, ranging from far below the average (1), lower than the average (2), the average (3), higher than the average (4), and far above the average (5). The self‐report questionnaire has the advantage of gathering information on a variety of facets and behaviors regarding spatial navigation ability in real environment. Previous studies have shown that one question on navigation ability can predict performances in spatial navigation tasks (Montello & Pick, 1993; Prestopnik & Roskos‐Ewoldson, 2000; Sholl, 1988; Sholl, Acacio, Makar, & Leon, 2000), and test‐retest reliability of one‐item scale is very high (average correlation of 0.93, Kozlowski & Bryant, 1977). Therefore, the one‐item scale provided a simple and valid measure of navigation ability.

#### 2.3.2 Santa Barbara Sense of Direction questionnaire

The Santa Barbara Sense of Direction (SBSOD) (Hegarty et al., 2002) is a standard questionnaire consisting of fifteen items on sense of direction in a large‐scale environment. Example items are “I very easily get lost in a new city” and “I can usually remember a new route after I have travelled it only once.” Participants were instructed to indicate the extent to which they agreed or disagreed with each statement in a 5‐point Likert‐type scale (Hund & Nazarczuk, 2009; Ventura, Shute, Wright, & Zhao, 2013). The total SBSOD score was used to index the participants’ sense of direction, with higher scores indicating better navigation ability. The SBSOD was administrated in Chinese, and the standard translation and blind back translation between English and Chinese were executed by four native Chinese speakers who had studied English for more than 10 years. The Chinese version of the SBSOD has been demonstrated to be a valid and reliable measure for navigation (Montello & Xiao, 2011). In this study, the Cronbach's α was 0.861 and Spearman‐Brown Coefficient was 0.858, suggesting that the Chinese version of the SBSOD had a good internal reliability.

### 2.4 Task‐state fMRI on scene perception

To characterize the functionality of the navigation‐related regions identified in the aforementioned VBM analysis, participants were instructed to passively view movie clips containing scenes, faces, objects, or scrambled objects. To simulate real‐life situations, the clips for the scene condition depicted leafy pastoral scenes shot from the window of a slow‐moving car and from films taken while flying through canyons or walking through tunnels. Faces, objects, and scrambled objects were included to examine the selectivity of the navigation‐related regions to visually presented scenes. The faces were those of playing children, the objects were moving toys, and the scrambled objects were constructed by scrambling each frame of the object movie clips (for more details on the stimuli, see Pitcher, Dilks, Saxe, Triantafyllou, & Kanwisher, 2011). Each stimulus category was presented in an 18‐s block that contained six 3‐s clips; four blocks with four stimulus categories constituted a consecutive block set. Each run contained two block sets, intermixed with three 18‐s rest blocks at the beginning, middle, and end of the run. There were three runs total, each of which lasted 3 min 18 s.

### 2.5 Resting‐state fMRI

To assess the neural network constituted by the navigation‐related regions during rest, resting‐state fMRI was performed when the participants were instructed to relax without engaging in any specific task and to remain still with their eyes closed. The total scan time was 8 min, which gave rise to a continuous time course consisting of 240 data points (TR = 2 s).

### 2.6 Data acquisition

The assessment of both general navigation ability and sense of direction was performed in the group session. The structural and resting‐state fMRI data were acquired in the same session, whereas the task‐state fMRI data were acquired about 1 year later. The MRI data were acquired on a Siemens 3T Trio scanner (MAGENTOM Trio, a Tim system) with a 12‐channel phased‐array head coil at the BNU Imaging Center for Brain Research, Beijing, China. T1‐weighted structure images were acquired with a magnetization‐prepared rapid gradient‐echo (MPRAGE) sequence (TR/TE/TI = 2.53 s/3.45 ms/1.1 sec, FA = 7 degrees, voxel size = 1 × 1×1 mm, slice thickness = 1.33 mm, number of volumes = 128) for each participant. The task‐state fMRI was acquired using a T2*‐weighted gradient‐echo echo‐planar‐imaging (GRE‐EPI) sequence (TR = 2000 ms, TE = 30 ms, flip angle = 90 degrees, number of slices = 30, voxel size = 3.125 × 3.125 × 4.8 mm). The rs‐fMRI was acquired using a T2*‐weighted GRE‐EPI sequence with different parameters from task‐state fMRI (TR = 2000 ms, TE = 30 ms, flip angle = 90 degrees, number of slices = 33, voxel size = 3.125 × 3.125 × 3.6 mm). Both behavioral and MRI data are available upon request.

### 2.7 Data analysis

#### 2.7.1 Neuroanatomical correlates of spatial navigation

VBM was employed to explore the neuroanatomical correlates of behavioral performance across participants (Ashburner & Friston, 2000). In this study, VBM was performed using SPM8 (Statistical Parametric Mapping, Wellcome Department of Imaging Neuroscience, London, UK, RRID: SCR_007037) and DARTEL (Wellcome Department of Imaging Neuroscience) on T1‐weighted structural images. First, image quality was assessed by manual visual inspection. Second, the origin of the brain was manually set to the anterior commissure for each participant. Third, images were segmented into four distinct tissue classes: gray matter (GM), white matter, cerebrospinal fluid, and everything else (e.g., skull and scalp) using a unified segmentation approach (Ashburner & Friston, 2005). Fourth, GM images for each participant were normalized to a study‐specific template in MNI152 space using the Diffeomorphic Anatomical Registration through Exponential Lie algebra (DARTEL) registration method (Ashburner, 2007). The DARTEL registration involves, repetitively, computing the study‐specific template based on the average tissue probability maps from all participants and then warping all participants’ tissue maps into the generated template to improve the alignment. Fifth, GM voxel values were modulated by multiplying the Jacobian determinants derived from the normalization to preserve the volume of tissue from each structure after warping. The modulated GM images were then smoothed with an 8‐mm full width at half maximum (FWHM) isotropic Gaussian kernel. Finally, to exclude noisy voxels, the modulated images were masked using an absolute masking with a threshold of 0.2. The masked‐modulated GM images were used for further statistical analyses.

Statistical analyses were performed using a general linear model (GLM). To investigate the neuroanatomical correlates of individual differences in general navigation ability, the statistical analysis treated age and gender as confounding covariates to account for age‐ and gender‐related anatomical variations, and the self‐report general navigational ability as the variable of interest. The false discovery rate (FDR) was set at *q* < 0.01 to correct the multiple comparisons at the voxel level across the brain.

Regions of interest (ROI) were defined as the intersection of the correlation map with the corresponding anatomical structure. Specifically, the retrosplenial complex (RSC) was defined as a continuous cluster in Brodmann 23, 29, 30, and 31 whose regional gray matter volume (rGMV) was significantly correlated with general navigation ability (right RSC, z > 3.9, corrected; left RSC, z > 3.7, corrected); the PHG, hippocampus (HPC), and thalamus (THAL) were defined by intersecting the correlation map with the parahippocampal gyrus, the lingual gyrus, the hippocampus, and the thalamus derived from the Harvard‐Oxford cortical/subcortical structural atlas (zs > 2.58, corrected); and the entorhinal cortex (EC) was defined by intersecting the correlation map with the entorhinal cortex derived from the Juelich historical atlas (z > 3.1, corrected). The probabilistic maps were thresholded at 25% for both the Harvard‐Oxford and Juelich historical atlases.

#### 2.7.2 Functionality of navigation‐related ROIs

fMRI data were analyzed with the fMRI Expert Analysis Tool (FEAT) of FSL (FMRIB's Software Library, http://www.fmrib.ox.ac.uk/fsl, RRID: SCR_002823). Preprocessing was performed with the default parameters of FEAT, consisting of motion correction, brain extraction, high‐pass temporal filtering (120 s cutoff) and spatial smoothing (with a Gaussian kernel of 5‐mm FWHM). Then, each run in a session was modeled separately for each participant. Statistical analyses on time series were performed with FILM (FMRIB's Improved Linear Model) with a local autocorrelation correction. A boxcar was convolved with a gamma hemodynamic response function, and its temporal derivative was used to model blood oxygen level‐dependent (BOLD) signal changes; the motion parameters from each run were entered into the model as confounding variables of no interest. Finally, the statistical image from each run was registered to each participant's high‐resolution structural image and then transformed to the standard MNI152 template using FLIRT (FMRIB's Linear Image Registration Tool).

To examine whether the navigation‐related ROIs responded selectively to the scenes, the MR signals were exacted from each predefined ROI of each participant. In addition, the selectivity for scenes of each ROI was calculated as the average of the *t* values of all voxels within an ROI with the contrast of scenes vs. faces and objects. Thus, the larger the *t* value, greater the degree of selectivity. Under the scene perception task, we also functionally defined the place‐selective TOS with the contrast of scenes vs. faces and objects (*p *< 10^−5^) (Dilks et al., 2013; Epstein, Higgins, & Thompson‐Schill, 2005).

#### 2.7.3 Resting‐state functional connectivity and clustering analysis

For each participant, to obtain stable resting‐state MR signals, the first four volumes were discarded. Then, the remaining 236 volumes were preprocessed with FSL. In addition to motion correction, spatial smoothing (FWHM = 6 mm), intensity normalization, the removal of linear trend, and several other preprocessing steps were used to reduce spurious variance unlikely to reflect neuronal activity. These steps included using a temporal band‐pass filter (0.01–0.1 Hz) to retain only low‐frequency signals (Cordes et al., 2001), regression of the time course obtained from rigid‐body and ‐head motion correction, the cerebrospinal fluid signal fluctuations were averaged from the cerebrospinal region, the white matter signal fluctuations averaged from the white matter region and the mean time course of whole‐brain BOLD fluctuations. Then, the 4‐D residual time series were registered to the standard MNI152 template using FLIRT.

The strength of FC was then used to characterize the functional synchronization among the ROIs and the hierarchical structure of the navigation network. First, a continuous time course for each ROI was extracted by averaging the time courses of all voxels in each of the predefined ROIs from the preprocessed images. Second, for each participant, a matrix on FC was created by calculating the Pearson correlation coefficient (*r*) between the time courses of each pair of ROIs. Third, we transformed the Pearson's correlation *r* values into Fisher's *z* values and the matrices were then averaged across participants. After averaging, the *z* values were transformed back to *r* values for further hierarchical clustering analyses. For the hierarchical clustering analysis, the complete‐linkage in the Python module (scipy.cluster.hierarchy) was applied to the averaged matrix to determine which pairs of ROIs were the most synchronized and which were the least synchronized (Eads, 2008). The value of “1– *r*” was used as an index for distance in the clustering. The resulting clusters, or dendrogram, were assessed by the cophenetic correlation coefficient, which is a measure of how faithfully the dendrogram represents the dissimilarities among observations. Specifically, the cophenetic correlation is defined as the linear correlation coefficient between original distances (i.e., dissimilarities) used to construct the dendrogram and cophenetic distances obtained from the dendrogram (i.e., the height of the link in the dendrogram at which observations are first joined). The more faithful the dendrogram, closer the cophenetic correlation coefficient is to one. The hierarchical clustering was considered successful if the cophenetic correlation coefficient was larger than 0.75.

We also examined whether the anatomical distance among the ROIs affected the hierarchical structure of the navigation network. The anatomical distance between each pair of ROIs was the Euclidean distance between the voxels that had a peak r value in the VBM analysis.

#### 2.7.4 Outlier participants

Two participants were excluded because their total score of SBSOD exceeded 2.5 standard deviations of the group mean. Another six participants were removed because of extraordinary scanner artifacts or abnormal brain structure (e.g., unusually large ventricles). Thus, 290 participants were included in the further analyses (age: 22–25; mean = 23.34, *SD* = 0.81, 120 males).

## 3 Results

There were considerable individual differences in the participants’ general navigation ability based on their self‐evaluation on the one‐item statement (Mean = 3.15, *SD* = 0.95; min = 1, max = 5). Kurtosis and the skewness of the participants’ scores fell within the range between −1 and +1 (*kurtosis* = −0.235, *skewness* = −0.374), indicating that the measurement was acceptable for the assumption of normality (Marcoulides & Hershberger, 1997). As expected, males (mean = 3.39, *SD* = 0.94) reported that they were better at navigation than females (mean = 2.98, *SD* = 0.93) (*t*_288_ = 3.69, *p *<* *.001), which was consistent with previous findings (Dabbs, Chang, Strong, & Milun, 1998; Hegarty, Montello, Richardson, Ishikawa, & Lovelace, 2006).

**Figure 1:**
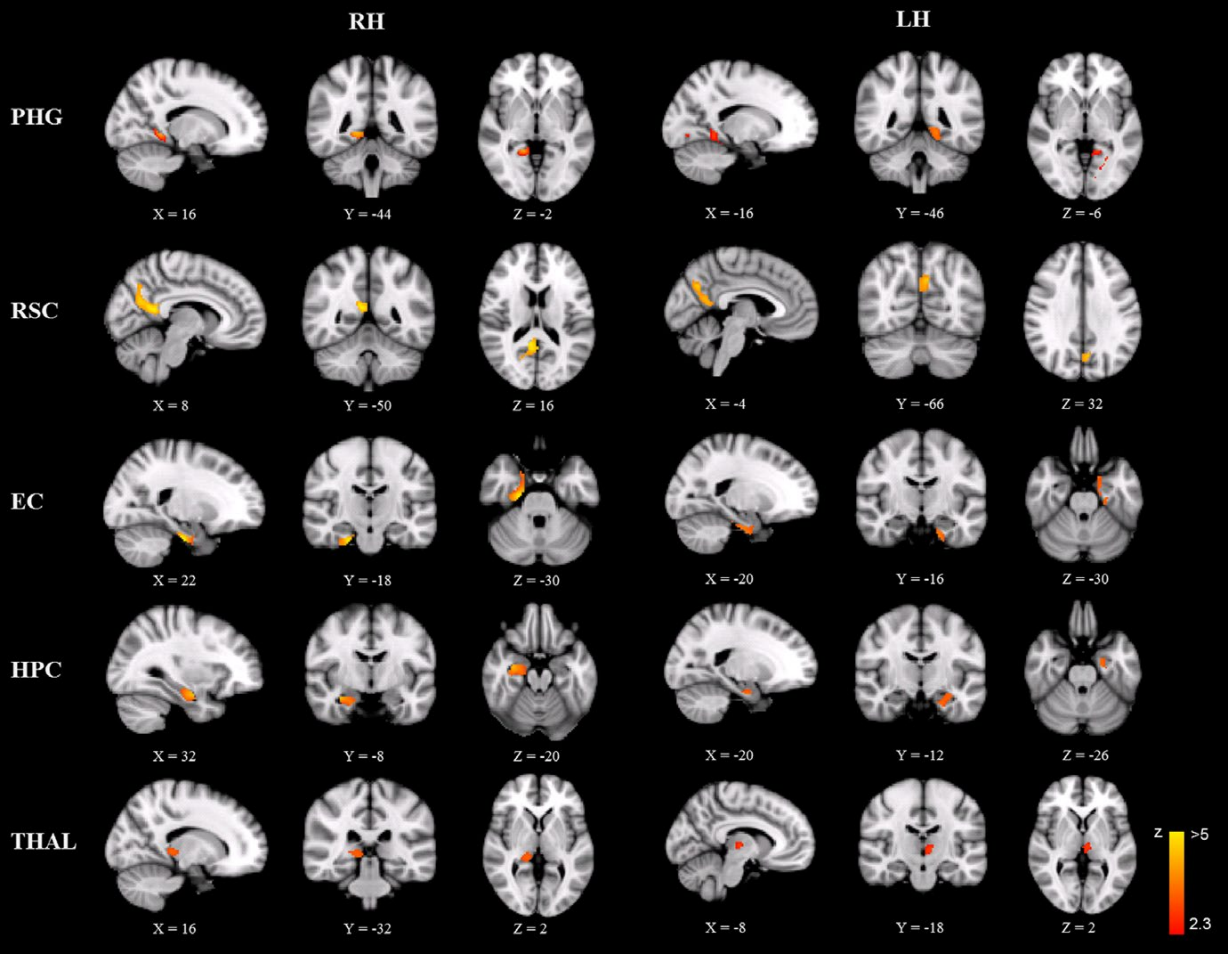
Neuroanatomical correlates of general navigation ability. The rGMV of the parahippocampus (PHG), retrosplenial complex (RSC), entorhinal cortex (EC), hippocampus (HPC), and thalamus (THAL) was positively correlated with general navigation ability. Peak voxel coordinates of each region are shown in the MNI stereotactic space. For all regions, *p *<* *.01, FDR corrected

To investigate the neuroanatomical correlates of general navigation ability, we correlated the GM volume of each voxel across the whole brain with the participants’ self‐report on the one‐item statement. Regions that showed a significant positive correlation between rGMV and general navigation ability are reported in Table 1 and Fig. S1. No significant negative correlation between rGMV and general navigation ability was found. Because previous studies have revealed the pivotal role of the medial temporal lobe and its surrounding in human spatial navigation (Aguirre, Detre, Alsop, & D'Esposito, 1996; Byrne, Becker, & Burgess, 2007; Kravitz, Saleem, Baker, & Mishkin, 2011; Maguire, 2001; Maguire et al., 1998; Marchette et al., 2014; Vann, Aggleton, & Maguire, 2009; Vass & Epstein, 2013), here we defined the navigation‐related regions in the medial temporal lobe and its surrounding as the ROIs and performed further analyses on them, including the bilateral PHG, RSC, EC, HPC, and THAL (all *p*s < .01, FDR corrected, Fig. 1). These results indicated that participants with larger rGMV of these regions had better navigation ability in daily life. A similar pattern of results was obtained with the total GMV regressed out (Fig. S2), indicating that our results could not be ascribed to the confounding effect of global signals. Although it was not the primary aim of this study, we did not observe reliable gender difference on the structure–behavior association in these navigation‐related regions—similar patterns of neuroanatomical correlates were observed in both male and female participants. Because there was no significant difference in the navigation ability‐rGMV correlation between each pair of the left‐ and right‐hemisphere ROIs (Steiger's Z‐test, all zs < 1), we pooled the data across hemispheres of each participant for further fMRI analyses.

**Table 1:** Regions with rGMV correlating with navigation ability

Region	MNI coordinate			Peak z‐value	*Peak p*‐value	Voxel number
	x	y	z			
R PHG	16	−44	−2	4.25	<.0001	123
L PHG	−16	−46	−6	3.56	.0004	194
R RSC	8	−50	16	4.78	<.0001	150
L RSC	−4	−66	32	4.4	<.0001	274
R HPC	32	−8	−20	4.17	<.0001	275
L HPC	−20	−12	−26	3.43	.0006	107
R EC	22	−18	−30	4.77	<.0001	244
L EC	−20	−16	−30	3.58	.0003	148
R THAL	16	−32	2	3.3	.001	84
L THAL	−8	−18	2	2.96	.0031	100
R OFG	36	−68	−16	4.39	<.0001	259
R ITG/temporal pole	42	6	−42	5.13	<.0001	337
L ITG/temporal pole	−40	6	−38	4.23	<.0001	130
R insular	36	−8	18	5.18	<.0001	517
L insular	−42	−10	20	4.6	<.0001	1045
R STG	62	−24	−10	5.14	<.0001	823
L STG	−58	−10	−4	4.89	<.0001	288
R MTG	56	−52	−4	5.45	<.0001	367

PHG, parahippocampus; RSC, retrosplenial complex; HPC, hippocampus; EC, entorhinal cortex; THAL, thalamus.

The false discovery rate (FDR) was set at *p* < .01 for multiple comparisons correction across the brain, and the corresponding threshold of uncorrected *p* values was *p* < .0038.

To validate the functionality of the ROIs defined by intersecting the regions related to the one‐item statement on general navigation ability with anatomically‐defined masks, we further examined whether the rGMV of these ROIs can predict the participants’ sense of direction measured by SBSOD, which is the core ability for spatial navigation (Kozlowski & Bryant, 1977). As expected, significant correlation was found between participants’ general navigation ability and the score of SBSOD (*r *=* *.78, *p *<* *.001). Importantly, the rGMVs of all of the navigation‐related regions identified above were correlated with participants’ sense of direction (PHG: *r *=* *.23, *p *<* *.001; RSC, *r *=* *.23, *p *<* *.001; EC, *r *=* *.19, *p *=* *.001; HPC, *r *=* *.18, *p *=* *.002; THAL, *r *=* *.19, *p *=* *.001), replicating the association between these ROIs and daily navigation ability. Thus, individual differences in navigation behaviors in daily life were partly accounted for by structural variations of these brain regions.

**Figure 2:**
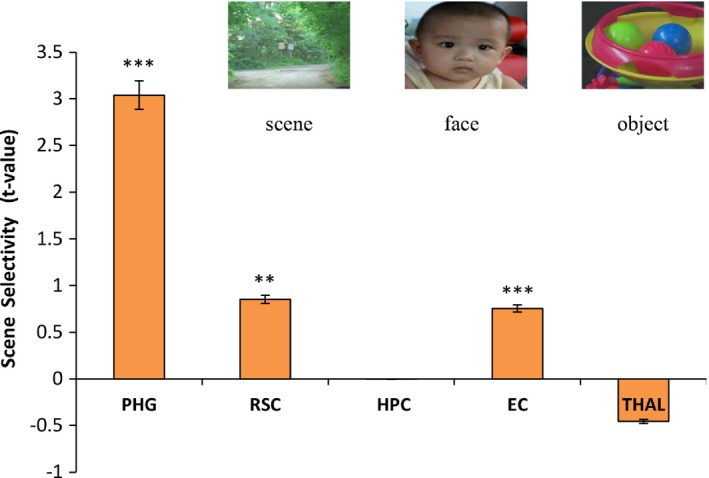
The scene selectivity [scenes > (objects + faces)] of the navigation‐related Regions of interest (ROI)s. The stimulus pictures were snapshots from movie clips. Note that the face shown here is not the actual face used in the experiment. The volunteer with this face has provided written consent for publication. The error bars indicate the standard error of the mean (*SEM*). *** indicates *p *<* *.001; ** indicates *p *<* *.01

Further visual inspection of the anatomical locations of the PHG and RSC suggests that they may correspond to the place‐selective regions identified in previous fMRI studies when participants performed a scene perception task. To test this anatomy‐function correspondence, we examined the fMRI responses of these regions during scene perception, which is a critical component for spatial navigation (Epstein & Vass, 2014; Wolbers & Hegarty, 2010), to preliminarily differentiate the functionality of the regions by examining whether they are involved in scene perception or other functional components of navigation instead. In the experiment, the participants perceived movie clips of natural scenes vs. faces, familiar objects, and scrambled objects. We found significant scene selectivity in the PHG (*t *=* *10.13, *p *<* *.001, Fig. 2) and RSC (*t *=* *2.90, *p *<* *.01), suggesting that the navigation‐related regions identified by the VBM analysis correspond to the parahippocampal place area (PPA) (Epstein & Kanwisher, 1998) and RSC (Bar & Aminoff, 2003; Maguire, 2001) reported in previous fMRI studies. Interestingly, the EC also showed significant scene selectivity (*t *=* *4.24, *p *<* *.001), which has been largely ignored in previous human fMRI studies. In contrast, the scene selectivity in the HPC and THAL was not significant (HPC: *t *=* *−0.02, *p *>* *.05; THAL: *t *=* *−2.03, *p *>* *.05), probably because they are involved in other components of spatial navigation than scene perception (Maguire et al., 1998; Morgan, Macevoy, Aguirre, & Epstein, 2011; Schinazi et al., 2013; Taube, Muller, & Ranck, 1990). In addition, none of the regions identified with the VBM analysis except the ROIs showed selective responses to scenes (Fig. S3), possibly because they are not specifically involved in navigation (Lambrey, Doeller, Berthoz, & Burgess, 2012; Rosenbaum, Ziegler, Winocur, Grady, & Moscovitch, 2004; Spiers, 2008). Note that under the scene perception task, we also identified the place‐selective transverse occipital sulcus (TOS) (Dilks et al., 2013; Epstein et al., 2005), which was not observed in the VBM analysis. To further examine the structure–behavior association of the TOS, we first functionally defined the TOS with the contrast of scenes vs. faces and objects and then correlated the mean rGMV of the TOS with the participants’ general navigation ability. As expected, there was no significant correlation between the rGMV of the TOS and spatial navigation ability (*r *=* *.29, *p *=* *.12).

The VBM analysis above identified multiple regions that are related to spatial navigation in daily life. Importantly, further fMRI studies on scene perception revealed the functional division of labor among these regions. Therefore, a critical question remains. How do these regions work collaboratively to constitute a neural network for spatial navigation? To address this question, we next examined the synchronization of spontaneous neural fluctuation among these regions to construct the neural network for spatial navigation.

**Figure 3:**
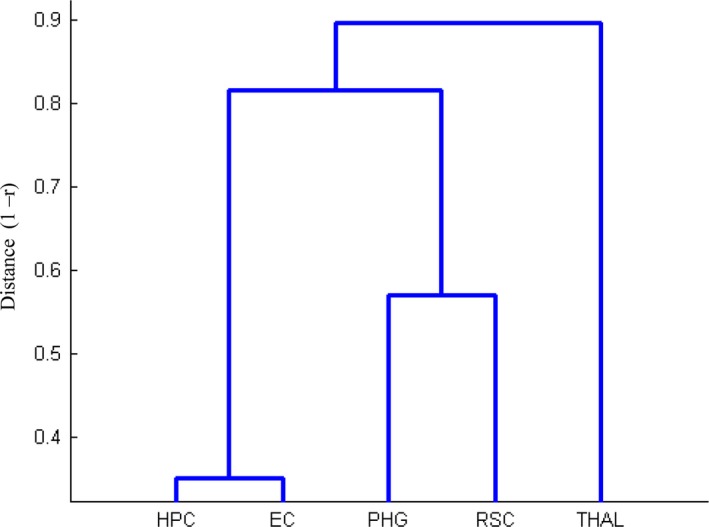
Hierarchically structured navigation‐related network. The dendrogram was obtained from the hierarchy clustering analysis based on the strength of functional connectivity among the VBM defined Regions of interest (ROI)s. The hippocampus (HPC) and entorhinal cortex (EC) constituted the component of the “cognitive map”, and the parahippocampus (PHG) and retrosplenial complex (RSC) constituted another component of “scene perception”. Thalamus (THAL) is more separated from the other two components and might be more involved in orientation processes

To do so, we acquired the participants’ continuous MR signals under resting state, and then calculated the FC in the navigation‐related regions. On average, the FC of all pairs of the navigation‐related regions was strong (PHG‐RSC: *r *=* *.41; PHG‐HPC: *r *=* *.39; PHG‐EC: *r *=* *.26; PHG‐THAL: *r *=* *.14; RSC‐HPC: *r *=* *.25; RSC‐THAL: *r *=* *.16; EC‐HPC: *r *=* *.62; HPC‐THAL: *r *=* *.14; EC‐THAL: *r *=* *.10; all *p*s < .001), indicating functional synchronization among these regions even without task engagement. However, the magnitude of the FCs differed greatly among the pairs of the regions, suggesting a hierarchical structure of the neural network constituted by these regions. Indeed, the hierarchical clustering analysis revealed that the navigation‐related regions were grouped into three relatively independent components of the navigation network (Cophenetic correlation coefficient = 0.88) (Fig. 3). The first component consisted of the PHG and the RSC, the second one contained the HPC and the EC, and the third one contained the THAL. In addition, the hierarchical clustering analysis revealed that the distance between the first two components was smaller, suggesting that the third component (i.e., the THAL) was more distinct in the navigation network. To further rule out the possibility that the hierarchical structure of the navigation network may result from anatomical distance among navigation‐related regions, we regressed out the anatomical distance between each region pair, and revealed a similar pattern of the hierarchical structure of the navigation network. Notably, the hierarchical clustering analysis containing all the regions identified with the VBM analysis revealed that they were grouped into two relatively independent components, with one component consisting of all the a priori defined ROIs, whereas the other consisted of the rest of the regions except the ROIs (Fig. S4). The relative independence between the two components confirmed the fMRI result that the regions except the ROIs may not be specifically involved in navigation (Lambrey et al., 2012; Rosenbaum et al., 2004; Spiers, 2008).

## 4 Discussion

In this study, we used the individual difference approach to investigate the neural correlates of spatial navigation in daily life. We found that the rGMV of a variety of brain regions, including the bilateral PHG, RSC, EC, HPC, and THAL, were correlated with the participants’ navigational ability in general, and the sense of direction in particular. Further fMRI study on scene perception showed that the navigation‐related regions identified in the VBM study, especially the PHG and RSC, also showed selective responses for scenes, consistent with previous fMRI studies on navigation (Bar & Aminoff, 2003; Epstein & Kanwisher, 1998). These results suggest the convergence of the anatomical structure and functional activation for spatial navigation. Importantly, these regions constituted a hierarchical neural network including three relatively independent components for navigation through synchronized neural fluctuations under a resting state. Taken together, our study linked the structural variance of a variety of cortical regions to daily experiences in spatial navigation, suggesting that successful spatial navigation relies on collaborative work from multiple regions to guide navigation behaviors.

Our study was consistent with a previous finding that good male navigators possess a larger HPC volume as compared to bad male navigators (Wegman et al., 2014), though the GMV differences related to navigation ability was not observed in females in their study, possibly because of a small sample size. However, the increased GMV in the HPC with navigation ability is not directly in line with the finding that the GMV increases with the length of time spent as a taxi driver in the posterior HPC and decreases in the anterior HPC (Maguire et al., 2000). It is probably due to that the length of time as a taxi driver reflects the duration of navigation training (Maguire et al., 2003, 2006; Woollett & Maguire, 2011), which is distinct from navigation ability as measured by SBSOD. Previous studies have revealed that the size of the HPC is correlated with the ability of flexibly using spatial knowledge and topographical representation (Hartley & Harlow, 2012; Schinazi et al., 2013), suggesting that the HPC may encode the cognitive map independent of viewpoints. Indeed, a recent study demonstrates that the HPC represents real‐world distances, which is the key feature of the cognitive map (Morgan et al., 2011). Another line of evidence comes from neurophysiological studies on rodents where the HPC contains place cells that encode information on locations of the environment (Ekstrom et al., 2003; O'Keefe & Dostrovsky, 1971). In addition to the HPC, our study also revealed a similar structure–behavior correlation in the EC, which contains grid cells that encode the contextual odometer information of our environment (Doeller, Barry, & Burgess, 2010; Fyhn, Molden, Witter, Moser, & Moser, 2004; Hafting, Fyhn, Molden, Moser, & Moser, 2005; Jacobs et al., 2013). Interestingly, the hierarchical clustering analysis revealed that the HPC and EC formed a relatively independent component of the navigation network, which is consistent with a recent optogenetic study indicating that the place cells in the HPC receive the output from the EC (Zhang et al., 2013). Combined with the fact that both the HPC and EC were not highly sensitive to visually presented scene stimuli, they may work collaboratively to form and represent the abstract cognitive map of the environment as a “cognitive map component” of the navigation network.

On the other hand, both the navigation‐related PHG and RSC responded more strongly to scenes compared to faces, objects, and scrambled objects, suggesting that they are involved in scene perception (Epstein & Kanwisher, 1998). The increased GMV in the PHG with navigation ability confirmed previous finding that good navigators showed more GMV in the PHG than poor navigators (Wegman et al., 2014). Indeed, previous fMRI studies have shown that the PHG is sensitive to the geometric layout of scenes (Epstein, Harris, Stanley, & Kanwisher, 1999; Epstein et al., 2005), and even non‐scene objects if they contain information for orientation (Troiani et al., 2012). Accordingly, patients with PHG lesions have difficulty recognizing large topographical entities such as streets, buildings, or intersections (Pallis, 1955). Similar to the PHG, the RSC is involved in processing scene‐relevant relationship between objects and their contexts (Bar, 2004), especially when viewing familiar locations in scenes (Vass & Epstein, 2013). A recent study further demonstrates that the RSC anchors to local topographical features and generalizes across local spatial contexts with similar geometric structures (Marchette et al., 2014). In addition, the RSC also helps translate between egocentric and allocentric spatial coding (Burgess, Becker, King, & O'Keefe, 2001). Taken together, the PHG is primarily involved in analyzing the geometric feature of the landmark, while the RSC is likely involved in determining one's position and orientation based on similar geometric structures in a large‐scale environment (Epstein & Vass, 2014; Marchette et al., 2014). Therefore, the functionality of the PHG and the RSC is complementary, and they likely team up to construct a coherent and detailed scene representation (Park & Chun, 2009). Not surprisingly, the hierarchical clustering analysis revealed that the PHG and RSC may form a “scene recognition component” of the navigation network, which may assist in extracting the geometric features of the local scene and integrating these features to determine one's location and orientation.

Finally, the THAL alone forms the third component, which is relatively independent from both the “cognitive map component” (i.e., the HPC and EC) and the “scene recognition component” (i.e., the PHG and RSC). Neurophysiological studies on rodents have revealed that the THAL contains head‐direction cells to encode egocentric spatial information (Taube et al., 1990), and fMRI studies of humans found that the THAL is activated during navigation in a virtual reality environment (Maguire et al., 1998). Though the exact function of the human THAL requires further study, it may provide information on the heading direction of locomotion for navigation (Taube, 2007).

In conclusion, our study links brain morphology and navigation ability, which may also shed light on the etiology of cognitive disorders related to spatial navigation (e.g., Alzheimer's disease, Topographical Disorientation Disorder). However, there are several limitations of this study. First, in our study, navigation ability was linked only to regional structural variation to reveal the neuroanatomical basis of navigation ability, and future studies are needed to link regional activation and FC between regions with navigation ability and examine the convergence among contributions of different imaging modalities. Second, we found that some regions identified by VBM (e.g., the PHG) corresponded to the regions identified by fMRI under navigation‐related tasks (e.g., the PPA), suggesting the convergence of the anatomical structure and functional activation of the navigation‐related regions, while the TOS did not exhibit such anatomical‐functional correspondence. The exact relationship between anatomical structure and functional activation needs to be illuminated in future research. Third, we found that the navigation‐related regions are functionally connected in resting‐state to form a hierarchical neural network for navigation. The three components of the network fit nicely with the three subsystem model of human navigation (i.e., path integration system, place recognition system, and reorientation system) proposed by Wang and Spelke (2002), and the three components in the model for landmark‐based piloting (i.e., landmark recognition, orientation, and cognitive map representation) proposed by Epstein and Vass (2014). It would be interesting to know how the network dynamically adjusts the connection weights among constituent nodes to adapt to a variety of navigation tasks (see also Zhen, Fang, & Liu, 2013). Finally, the HPC and RSC, which are key nodes of navigation network, have also been implicated in other processes, including default mode, episodic memory, and theory of mind (Buckner & Carroll, 2007; Hassabis & Maguire, 2007; Spreng, Mar, & Kim, 2009). Future studies are needed to investigate how these regions flexibly switch between different functions by interacting dynamically with other regions in corresponding networks.

## Conflict of Interest

The authors declare no competing financial interests.
