# Supplementary material for: Structural and functional neural correlates of spatial navigation: a combined voxel‐based morphometry and functional connectivity study
Source: Brain Behav. 2016 Oct 3;6(12):e00572. doi: 10.1002/brb3.572 (PMC5166998; doi:10.1002/brb3.572)
Supplement: Supplementary file 1 [file BRB3-6-e00572-s001.docx]

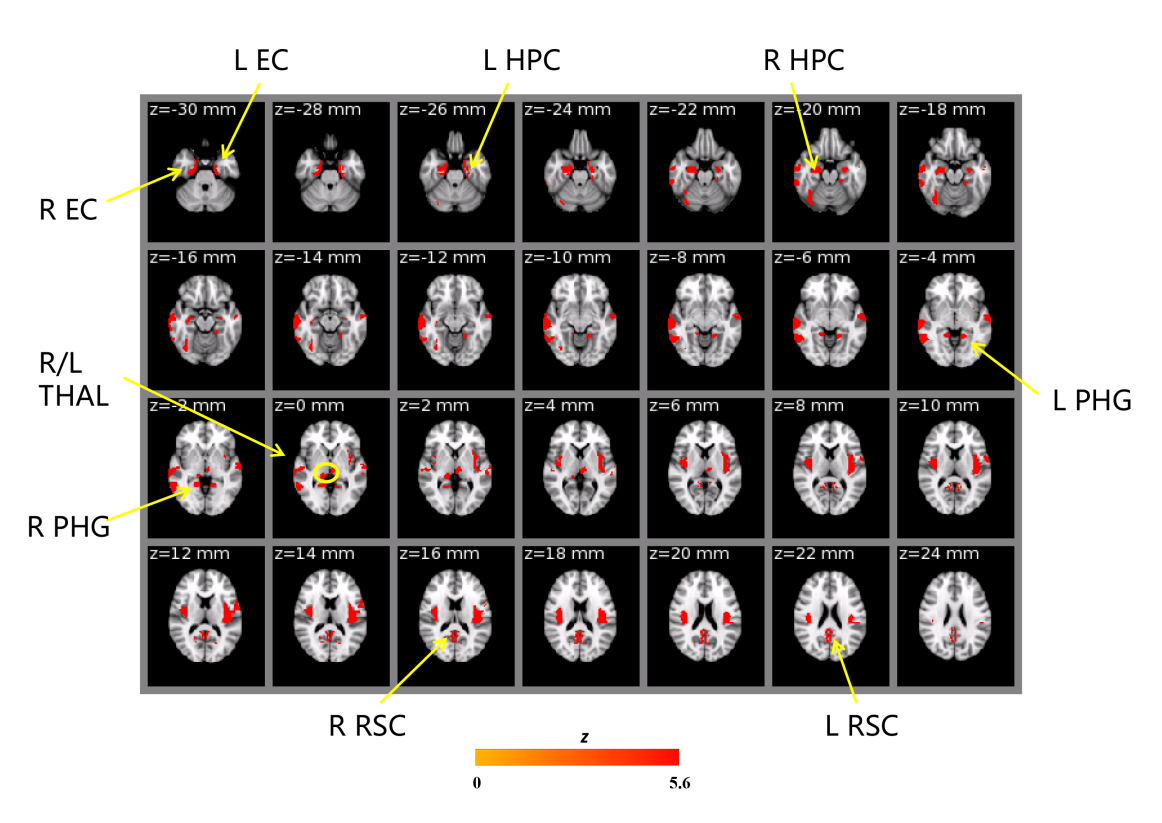


**Supplementary Figure 1.** **Regions across the brain where the GMV positively correlated with general navigation ability** (*p* < 0.01, corrected for FDR). No significant negative correlation was found.

**
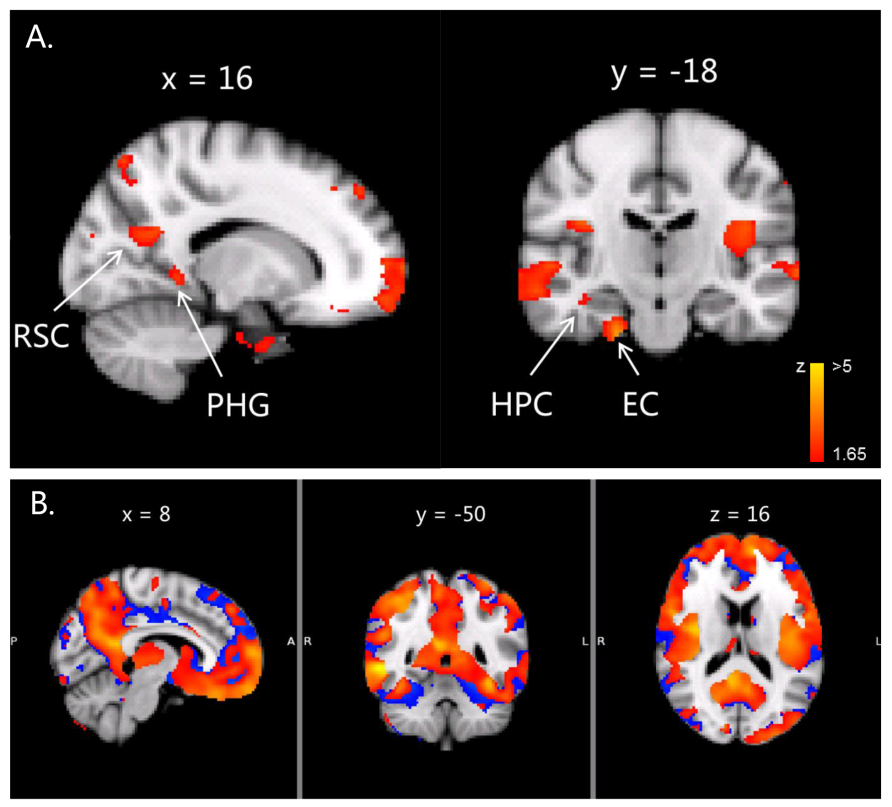
**

**Supplementary Figure 2**. **Neuroanatomical correlates of general navigation ability after regressing out the total gray matter volume (GMV).** A) Regions in which variability in rGMV exhibited significant positive correlation with general navigation ability; no negative correlations were found (corrected, p<0.01). B) The overlap between the uncorrected results before (blue) and after regressing out the total GMV (red).

**
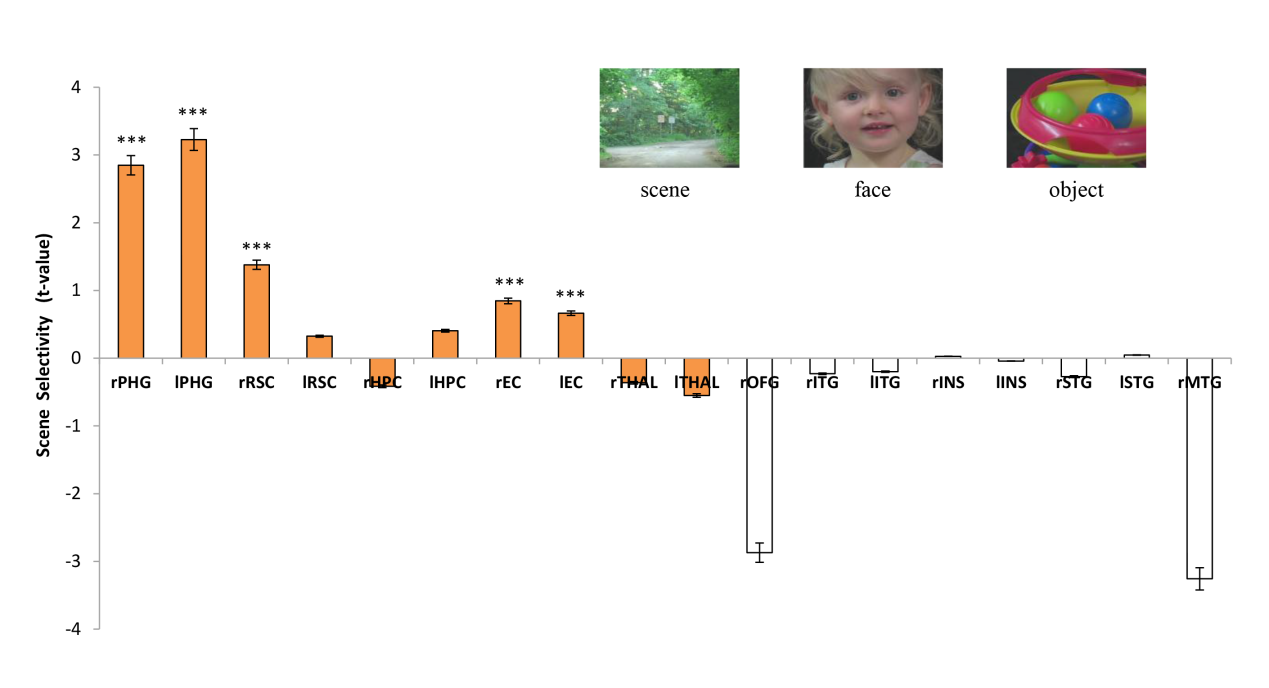
**

**Supplementary Figure 3. The scene selectivity [scenes > (objects + faces)] of all navigation-related regions identified in the VBM analysis.** The a priori defined ROIs that have been extensively examined in the main text are shown in orange, and the regions outside the ROIs are shown in white. *** indicates p < 0.001.

**
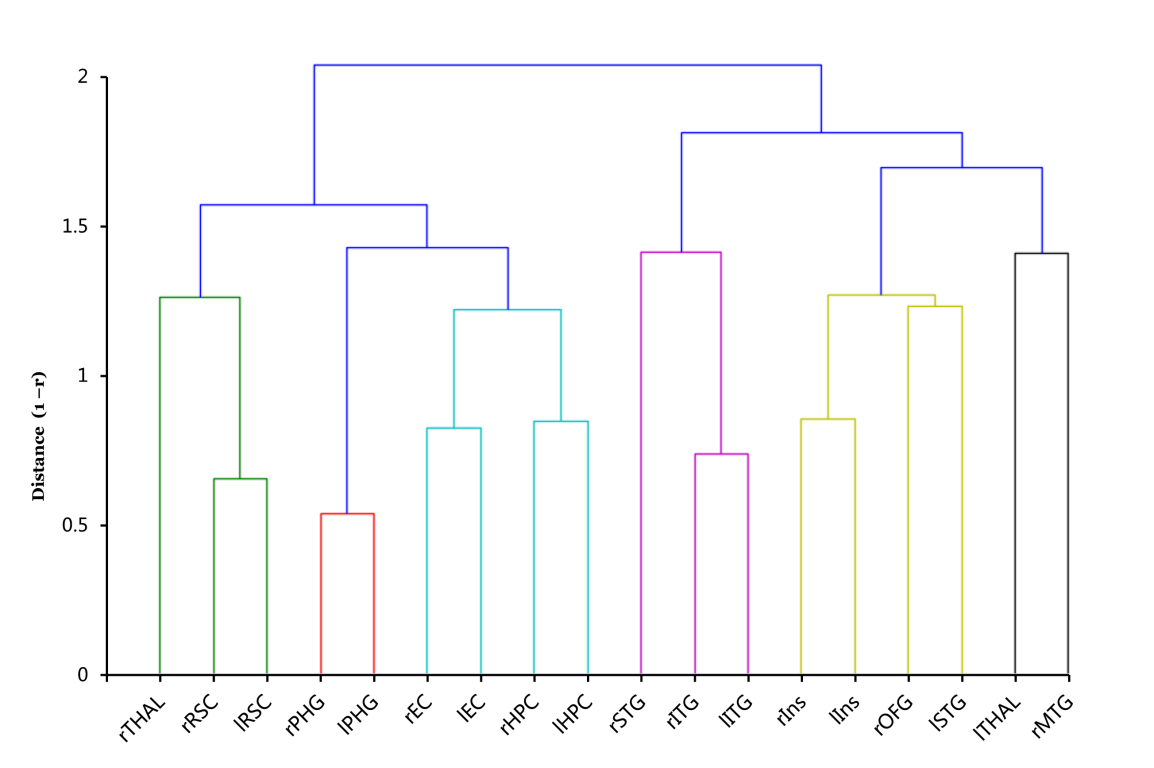
**

**Supplementary Figure 4**. **The hierarchical structure of the network consisted of all navigation-related regions identified in the VBM analysis.** We performed a hierarchical clustering analysis containing all the regions identified in the VBM analysis. Each ROI was defined as a 5mm radius sphere around each peak from the VBM analysis. The dendrogram revealed that all the navigation-related regions were grouped into two relatively independent components (Cophenetic correlation coefficient = 0.73), with the left cluster of the dendrogram consisting of all the a priori defined ROIs, whereas the right cluster consisting of all the regions outside the ROIs.
